# Co-Existence of Hypertensive and Anti-Hypertensive Constituents, Synephrine, and Nobiletin in *Citrus unshiu* Peel

**DOI:** 10.3390/molecules24071197

**Published:** 2019-03-27

**Authors:** Jung-Joon Kim, Keunyoung Kim, Ye-Ryeon Jung, Yiying Bian, Thien Ngo, Ok-Nam Bae, Kyung-Min Lim, Jin-Ho Chung

**Affiliations:** 1 College of Pharmacy, Seoul National University, Seoul 08826, Korea; jj128kim@korea.kr (J.-J.K.), millio1014@naver.com (K.K.), jyr074@naver.com (Y.-R.J.), byy19900719@hotmail.com (Y.B.), ngothien86@gmail.com (T.N.); 2 College of Pharmacy, Hanyang University, Ansan 15588, Korea; onbae@hanyang.ac.kr; 3 College of Pharmacy, Ewha Womans University, Seoul 03760, Korea

**Keywords:** synephrine, nobiletin, *Citrus unshiu*, vasoconstriction, hypertension, bioassay

## Abstract

A single herb can contain multiple constituents with diverse bioactivities. We found that the extract of *Citrus unshiu* peel (CUP), induced abnormal vasoconstriction responses on the freshly isolated rat aortic rings in vitro. CUP stimulated the vasoconstriction alone, and it suppressed the phenylephrine-stimulated vasoconstriction. We studied the reasons behind this abnormal vasoconstriction pattern. Major constituents of CUP were determined and evaluated for their vaso-activities. Notably, synephrine, a contractile agonist, and nobiletin, newly identified to have anti-contractile activity co-existed in CUP. Synephrine and nobiletin competitively blocked or activated the same contractile targets resulting in contradicting and abnormal vasoconstriction responses. Accordingly, the vasoconstriction pattern varies significantly depending on the relative contents of synephrine and nobiletin in CUP. Interestingly, this response pattern could be observed with another plant extract, *Acorus gramineus Sol.* Collectively, we demonstrated that active ingredients with contradicting bioactivities could co-exist in a single plant extract, interact and produce abnormal response patterns in bioassay, which would give an important insight into the interpretation of unusual activity patterns induced by plant extracts.

## 1. Introduction

Plants have many beneficial effects on human health as well as providing food or fiber [1]. Also, the safety of plants is generally well-established since they are used for food or cosmetics [2] without significant side effects. Accordingly, extracts of plants commonly constituting a major part of the natural product library for the initial search of biologically active compounds for the development of plant-based dietary supplements, cosmetics [3], nutraceuticals [4] or therapeutic drugs [5,6].

Generally, bioactivity of a substance is screened through bioassays that are composed of a pre-incubation step with a test substance, and the addition of stimuli to initiate biological responses of interest [7]. Incidentally, in bioassays for plant extracts, abnormal response patterns or inconsistency in potency or efficacy of activity are often observed [8]. This phenomenon is quite common, but in most cases, the reasons remain unknown, since there are multiple bioactive constituents even in a single plant extract, and accordingly, many more interactions between them occur, that are difficult to elucidate. Indeed synergy and antagonism between constituents of plant extract have been a focus of recent reviews [8,9], since the information could be helpful for the development of standardized, effect-optimized mono- and multi-extract preparations for dietary supplements or botanical drugs.

*Citrus unshiu* is a seedless citrus fruit which is widely consumed in Korea, Japan, and China [10]. Its dried peels (*Citrus unshiu* peel, CUP) are used as a tea to improve respiratory distresses or blood circulation [11]. CUP is well-known to be enriched with flavonoids and contains various active components like hesperidin, naringin, narirutin, and neohesperidin [12,13,14]. Beneficial health effects of CUP, such as anti-oxidant [14], anti-cancer, anti-inflammatory [15,16], hypoglycemic [10], lipolytic [17,18], and cardiovascular protective activities have been reported, to which those flavonoids may be attributable. However, the effects of CUP on the vasoconstriction, an important therapeutic target for hypertension [19], and critical risk factor for other cardiovascular diseases [20], are not well-illustrated.

Here we examined the anti-contractile effects of CUP, along with other plant extracts employing in vitro agonist-induced vasoconstriction model, freshly isolated rat aortic rings, a commonly used functional assay for anti-hypertensive activities [21]. Notably, CUP induced an abnormal vasoconstriction-pattern, i.e., the induction of vasoconstriction alone during the pre-incubation period, and suppression of agonist-induced vasoconstriction after the addition of phenylephrine (PE). To clarify the reason behind this, we identified the flavonoid constituents of CUP through high-performance liquid chromatography (HPLC) analysis and examined their activities, and contributions on the effects of CUP on vasoconstriction, in order to provide important insights to understand the bioactivities of plant extracts.

## 2. Results and Discussion

**Figure 1:**
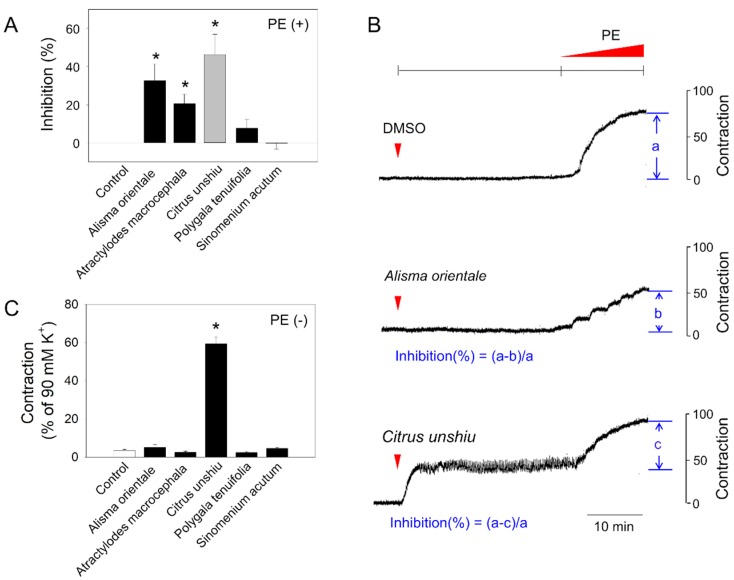
Effects of plant extracts on phenylephrine (PE)-induced vasoconstriction in aortic rings. After 250 μg/mL of extracts were treated to aortic rings for 30 min, PE-induced vasoconstriction was measured. (**A**) Inhibitory effects of plant extracts on PE-induced vasoconstriction. (**B**) Representative tracing of dimethylsulfoxide (DMSO) (vehicle), *Alisma orientale* and *Citrus unshiu* extract. (**C**) Contraction by herbal extracts in the absence of PE. Values are mean ± SEM of three to four independent experiments. *, significant differences from the control (*p* < 0.05).

The bioactivity screening for anti-contractile effects was composed of 30 min pre-incubation period with the addition of a test substance onto freshly isolated rat aorta in organ bath system, and contraction was initiated with PE. As shown in Figure 1A, 3 of 5 tested plant extracts (*Alisma orientale*, *Atractylodes macrocephala*, and *Citrus unshiu*) displayed anti-contractile effects. However, the pattern was not identical, i.e., while *Alisma orientale* manifested pure antagonistic effects, those of CUP was more complicated (Figure 1B). Inhibition (%), which is calculated based on the effect size of PE-induced contraction, suggested that the anti-contractile effect of CUP was substantial, but when the contraction prompted during pre-incubation of CUP (Figure 1C), was excluded, the inhibitory effect was estimated to be negligible, resulting in a false positive.

**Figure 2:**
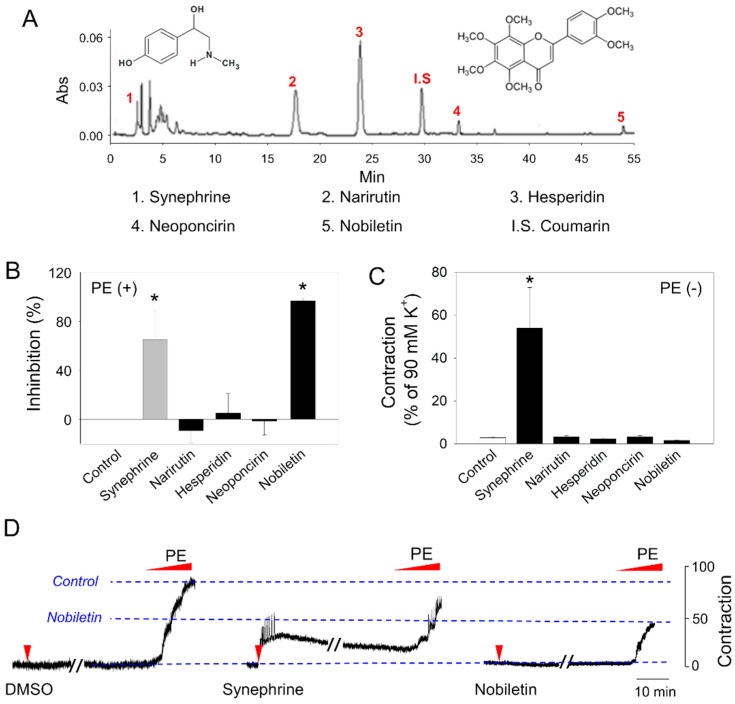
Effects of major *Citrus unshiu* components on phenylephrine (PE)-induced vasoconstriction in aortic rings. (**A**) High-performance liquid chromatography chromatogram of 5 major components of *Citrus unshiu*. Coumarin was used as an internal standard. After 100 μM of each component was treated to aortic rings for 30 min, PE-induced vasoconstriction was determined. (**B**) Inhibition of PE-induced contraction and (**C**) contraction in the absence of PE by 5 major components. (**D**) Representative tracings of DMSO (vehicle), synephrine and nobiletin are shown. Values are mean ± SEM of three independent experiments. *, significant differences from the control (*p* < 0.05).

To clarify the reason behind this, we characterized the 5 major components of CUP through HPLC and authentic standards, and evaluated their respective effects on basal tonicity or agonist-induced vasoconstriction. In CUP, synephrine, neoponcirin, narirutin, nobiletin, and hesperidin were identified (Figure 2A). Of these 5 components, synephrine and nobiletin appeared to exhibit inhibitory effects (Figure 2B) but synephrine-induced contraction by itself without PE (Figure 2C). This pattern could be visually confirmed again in the tracing data (Figure 2D), which was comparable to CUP in Figure 1B, suggesting that the mixed contribution of an agonistic synephrine and an antagonistic nobiletin resulted in the unusual response pattern of CUP.

**Figure 3:**
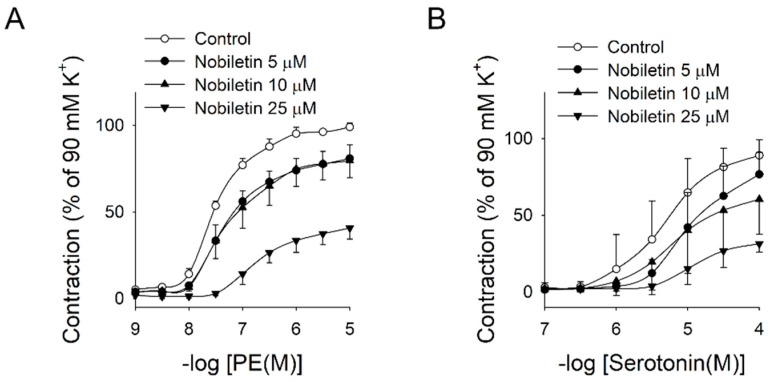

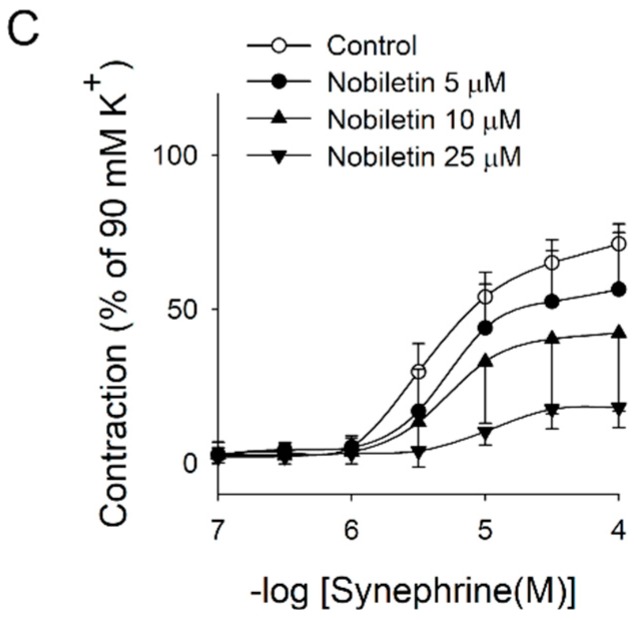
Effects of nobiletin on agonist-induced vasoconstriction in aortic rings. Aortic rings were pretreated with various concentrations of nobiletin for 30 min, then vasoconstriction by agonists ((**A**): PE, (**B**): serotonin, (**C**): synephrine) was examined. Values are mean ± SEM of four independent experiments.

The contractile effects of synephrine have been already reported [22], which is mediated via the activation of adrenergic α1-receptors and serotonergic (5-HT_1D_ and 5-HT_2A_) receptors [23]. Nobiletin is known to possess anti-oxidant, anti-viral [24], anti-inflammatory, antitumor [25], anti-thrombotic [26], hypoglycemic [27], lipolytic [28] and antiproliferative activities [29]. Notably, nobiletin showed anti-constrictive effects in the gastrointestinal tract [30,31]. Ikemura et al. [26] also provided a clue for anti-hypertensive effects of nobiletin by feeding experiments in SHR rats, but the results were largely observational lacking in mechanistic explanation. Recently, Yang et al. reported that nobiletin exhibits relaxation in mesenteric arteries through the calcium-eNOS pathway in endothelium [32]. However, the anti-contractile effect of nobiletin against vasoconstriction has not been reported, to the best of our knowledge. To elucidate the anti-contractile effects of nobiletin, we examined the concentration-dependent effects of nobiletin on PE and serotonin-induced vasoconstriction. As shown in Figure 3A,B, nobiletin inhibited PE and serotonin-induced vasoconstriction significantly in a concentration-dependent manner, reflecting that nobiletin can modulate vasoconstriction via adrenergic and serotonergic pathways. Nobiletin could also attenuate synephrine-induced vasoconstriction (Figure 3C), indicating that they can act mutually antagonistic, rendering the pattern of responses to CUP incongruous and highly variable.

**Figure 4:**
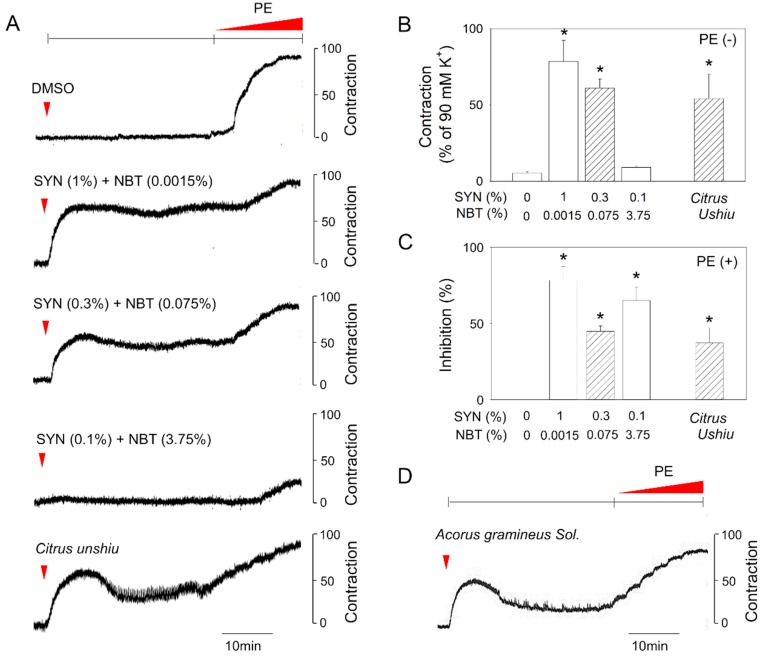
Response patterns of vasoconstriction by varying relative ratios of synephrine (SYN) and nobiletin (NBT). After aortic rings were pretreated with a mixture of SYN and NBT for 30 min, PE-induced vasoconstriction was determined. (**A**) Representative tracing, (**B**) contractile effects in the absence of PE, and (**C**) inhibitory effects on PE-induced vasoconstriction by varying relative ratios of SYN and NBT and *Citrus unshiu* extract. (**D**) Representative tracing of *Acorus gramineus Sol.* extract. Values are mean ± SEM of three to four independent experiments. *, significant differences from the control (*p* < 0.05).

Indeed, we observed that different response patterns were induced by varying the relative ratios of synephrine and nobiletin (Figure 4A). Of note, the combined treatment of synephrine 0.3% and nobiletin 0.075% as determined in CUP, manifested a similar response pattern to that of CUP on basal tonicity and PE-induced contraction in rat aortic ring (Figure 4A–C). A higher ratio of synephrine or nobiletin resulted in apparently pure agonistic or antagonistic effect respectively, signifying that combined effects of synephrine and nobiletin may explain abnormal response patterns of CUP. The variable relative ratios of synephrine and nobiletin might also explain the inconsistent potency or efficacy of CUPs from different sources although further studies are necessary to confirm the correlation of functional assays and the relative contents of synephrine and nobiletin in CUP.

Importantly, this phenomenon was not limited to CUP. Another herbal extract, *Acorus gramineus Sol.* also induced a response pattern similar to that of CUP, namely, triggering contractions without PE and suppression of PE-induced contraction (Figure 4D), reflecting that the co-existence of active components with agonistic and antagonistic activities in a single plant extract is not uncommon. With this result, we could suggest that plant extracts showing an abnormal response pattern in bioactivity assays is revisited to identify the co-existence of active constituents. Through this research, we believe that further diversity of active components can be newly re-discovered for plant extracts and mixed responses can be understood.

## 3. Materials and Methods

### 3.1. Reagents

Phenylephrine (PE), serotonin creatinine sulfate, dimethylsulfoxide (DMSO), and synephrine, nobiletin were purchased from Sigma Chemical Co. (St. Louis, MO, USA). All other reagents used were of the highest purity available.

### 3.2. Extraction and Isolation

The herbal extracts and five major components, isolated from *Citrus unshiu,* were provided from the project team of Superfund on ‘Identification of the Efficacy of Biologically Active Components from Oriental Herbal Medicines’ supported by the Korea Food and Drug Administration. In brief, the dry powder of herbs (*Alisma orientale*, *Atractylodes macrocephala*, *Citrus unshiu*, *Polygala tenuifolia*, *Sinomenium acutum*, *Acorus gramineus Solander*) were extracted with 70% ethanol at 70–80 °C for 3 hr. The extraction was repeated three times. After filtration and concentration under reduced pressure, the extracts were lyophilized, and the resultant powder was stored at −20 °C. For in vitro experiments, herbal extracts were dissolved in DMSO before use. Major components of *Citrus unshiu* extract were identified using HPLC system (Waters Corp., Milford, CT, USA) was equipped with Waters 2695 separation module with Waters 996 photodiode array detector, Waters 600 pump controller, Waters 717 autosampler and Waters 486 tunable absorbance detector. HPLC analysis was conducted using YMC-J’sphere ODS-H80 (4.6 × 250 mm, 4 μm; YMC, Kyoto, Japan) column, and the column temperature was maintained at 25 °C. Gradient flows for the two solvent system (solvent A, 0.1% phosphoric acid in acetonitrile; solvent B, 0.1% phosphoric acid in water) were as follows: 0 min (A:B = 18:82), 20 min (A:B = 18:82), 60 min (A:B = 55:45). The mobile phase flow rate was 1 mL/min. The chromatogram was monitored at 280 nm.

### 3.3. Animals

The entire animal protocol was approved by the Ethics Committee of Animal Service Center at Seoul National University. Male Sprague-Dawley rats (SamTako, Seoul, Korea) weighing 250–300 g (8 to 9 weeks old). Before the experiments, animals were acclimated for one week in the laboratory animal facility maintained at constant temperature (22 ± 2 °C) and humidity (50 ± 5%) with a 12-hr light/dark cycle. Food (Cargill Agri Purina, Inc., Seongnam, Korea) and water were provided *ad libitum*.

### 3.4. Measurement of Vasoconstriction in Isolated Aortic Rings

After rats were decapitated to exsanguinate, the thoracic aorta was carefully isolated and cut into ring segments in lengths of 3–4 mm on ice. The rings were then mounted on organ baths filled with Krebs-Ringer solution (115.5 mM NaCl, 4.6 mM KCl, 1.2 mM KH_2_PO_4_, 1.2 mM MgSO_4_, 2.5 mM CaCl_2_, 25 mM NaHCO_3_, and 11.1 mM glucose, pH 7.4) continuously saturated with 95% O_2_/5% CO_2_ mixture gas and maintained at 37 °C. The change in tension was measured with Grass FT03 force transducers (Grass Instrument Co., Quincy, MA, USA) and recorded using the AcqKnowledge III (BIOPAC Systems Inc., Goleta, CA, USA). To investigate the effect on vasoconstriction, the aortic rings were treated with herb extracts or components, and vasoconstriction was initiated by the cumulative addition of PE, serotonin, or synephrine.

### 3.5. Statistical Analysis

The means and standard errors of means were calculated for all treatment groups. The data were subjected to Students *t*-test using SPSS software (SPSS Inc., Chicago, IL, USA) to determine which means were significantly different from the control.
